# Cytoprotective Role of Nrf2 in Electrical Pulse Stimulated C2C12 Myotube

**DOI:** 10.1371/journal.pone.0144835

**Published:** 2015-12-14

**Authors:** Masaki Horie, Eiji Warabi, Shoichi Komine, Sechang Oh, Junichi Shoda

**Affiliations:** 1 Division of Medical Science, Faculty of Medicine, University of Tsukuba, Tsukuba, Ibaraki, Japan; 2 Japan Society for the Promotion of Science, Tokyo, Japan; 3 Division of Biomedical Sciences, Faculty of Medicine, University of Tsukuba, Tsukuba, Ibaraki, Japan; 4 Graduate School of Comprehensive Human Sciences Majors of Medical Sciences, University of Tsukuba, Tsukuba, Ibaraki, Japan; University of Debrecen, HUNGARY

## Abstract

Regular physical exercise is central to a healthy lifestyle. However, exercise-related muscle contraction can induce reactive oxygen species and reactive nitrogen species (ROS/RNS) production in skeletal muscle. The nuclear factor-E2-related factor-2 (Nrf2) transcription factor is a cellular sensor for oxidative stress. Regulation of nuclear Nrf2 signaling regulates antioxidant responses and protects organ structure and function. However, the role of Nrf2 in exercise- or contraction-induced ROS/RNS production in skeletal muscle is not clear. In this study, using differentiated C2C12 cells and electrical pulse stimulation (EPS) of muscle contraction, we explored whether Nrf2 plays a role in the skeletal muscle response to muscle contraction-induced ROS/RNS. We found that EPS (40 V, 1 Hz, 2 ms) stimulated ROS/RNS accumulation and Nrf2 activation. We also showed that expression of NQO1, HO-1 and GCLM increased after EPS-induced muscle contraction and was remarkably suppressed in cells with Nrf2 knockdown. We also found that the antioxidant N-acetylcysteine (NAC) significantly attenuated Nrf2 activation after EPS, whereas the nitric oxide synthetase inhibitor Nω-nitro-L-arginine methyl ester (L-NAME) did not. Furthermore, Nrf2 knockdown after EPS markedly decreased ROS/RNS redox potential and cell viability and increased expression of the apoptosis marker Annexin V in C2C12 myotubes. These results indicate that Nrf2 activation and expression of Nrf2 regulated-genes protected muscle against the increased ROS caused by EPS-induced muscle contraction. Thus, our findings suggest that Nrf2 may be a key factor for preservation of muscle function during muscle contraction.

## Introduction

The transcription factor nuclear factor-E2-related factor-2 (Nrf2) serves as a cellular sensor for oxidative stress [1]. Nrf2 is sequestered in the cytosol by Kelch-like Ech-associated protein (Keap1) [2]. During an oxidative challenge, modification of Keap1 sulfhydryl groups results in the stabilization and nuclear translocation of Nrf2 [2,3]. Nrf2 plays a crucial role in regulating induction of antioxidant and other oxidative stress response genes by the antioxidant responsive element/electrophile-responsive element (ARE/EpRE) [3]. Activation of these genes serves to decrease the oxidative burden of a cell [1]. Regulation of Nrf2 nuclear signaling, therefore, preserves redox homeostasis and protects organ structure and function [1].

Regular physical exercise has many health benefits, including reduced risk of cardiovascular disease, cancer and diabetes [4,5]. However, exercise-related muscle contraction is also known to induce reactive oxygen species and reactive nitrogen species (ROS/RNS) in skeletal muscle [6]. It is clear from various reports that contracting skeletal muscles generate ROS/RNS and intense exercise can result in oxidative damage to cellular constituents [7,8]. Skeletal muscle generated ROS/RNS at rest, and these levels increased during contractile activity [7–10]. Production of ROS/RNS stimulated redox-sensitive signaling pathways that modify the cellular content of cytoprotective regulatory proteins such as superoxide dismutases (SODs) and catalase, and prevented oxidative damage to tissues [11]. In addition, exercise training and continued muscle contraction improved the antioxidant capacity of skeletal muscle, as evidenced by increased activity of glutathione peroxidase (GPx), SOD1 and SOD2 in muscle after training [11]. These previous studies suggested that antioxidant responses to oxidative stress are key factors to maintain muscle function during normal exercise and for muscle adaptation during intense exercise. However, the molecular mechanisms of muscle responses to oxidative stress during exercise remain poorly understood.

We hypothesized that ROS/RNS production during muscle contraction would induce Nrf2 activation and, thereby, activate Nrf2-mediated responses to oxidative stress, increasing the antioxidant capacity of skeletal muscle. However, previous studies have not identified a function for Nrf2 in exercise- or contraction-induced ROS/RNS in skeletal muscle. Therefore, using differentiated C2C12 skeletal muscle cells (myotubes) and electrical pulse stimulation (EPS), we investigated Nrf2 involvement in the adaptive response to oxidative stress occurring in skeletal muscle cells after EPS-induced contractile activity.

## Materials and Methods

### Reagents and Cell Culture

C2C12 skeletal muscle cells were obtained from the Cell Bank Institute, RIKEN BioResource Center (Ibaraki, Japan). The cells were cultured in growth medium (Dulbecco’s modified Eagle’s medium (DMEM)) containing 10% fetal bovine serum (FBS) and 1% penicillin and streptomycin solution in a humidified incubator at 37°C with 5% CO_2_. When C2C12 myoblast cultures reached confluence, they were switched to DMEM containing 2% heat-inactivated horse serum supplement (differentiation medium) for five days, and the C2C12 skeletal muscle cells differentiated into myotubes.

### Electrical pulse Stimulation Protocol

C2C12 cells were grown in 6-well plates. After differentiation, myotubes were stimulated using an electrical pulse stimulator (C-Pace EP; IonOptix, MA, USA). The EPS stimulation pulse was set at 2 ms with 14, 20, and 40 V and a frequency of 1 Hz. The electrical stimulation apparatus was modified from a previous design [12]. In all experiments, EPS treatment was performed for 1, 3 or 6 hours and cell extracts obtained from each dish were prepared immediately after EPS.

### Measurement of ROS/RNS

Intracellular ROS/RNS production was measured using 2′,7′-dichlorofluorescein diacetate (DCFH-DA). First, 2 μM DCFH-DA was added to the medium of EPS-treated or untreated myotubes. The samples were then incubated for 15 min in the dark at room temperature. Subsequently, cells were washed three times with culture medium. The DCFH-DA treated cells were evaluated under light microscopy, and microphotographs were taken with a digital camera (Olympus IX71; Olympus, Tokyo, Japan) attached to a microscope (Olympus DP71; Olympus). The digitally captured images were processed and analyzed using image analysis software (Image J; National Institutes of Health, MD, USA). Using Image J, DCFH-DA intensity was measured in 20 high-power fields (HPF) from 5 sections prepared from a cell cultured dish for each group.

### siRNA transfection

The siRNA target sequence for Nrf2 was 5'-GCA UGU UAC GUG AUG AGG AUG GAA A-3'. siRNA (80 nM) was transfected into C2C12 with Lipofectamine RNAiMAX Transfection Reagent (Invitrogen, CA, USA) according to the manufacturer’s instructions. Differentiation medium was replaced with Opti-MEM containing a mixture of siRNA and lipofectamine. The differentiated cells were cultured for another 24 hours and then used for experiments. Additionally, an siRNA negative control (mock; 80 nM) (Invitrogen) was transfected. There was almost no visible damage due to the transfection procedure.

### Chemical treatment

N-acetylcysteine (NAC) was from Wako (Wako, Tokyo, Japan), and Nω-nitro-L-arginine methyl ester hydrochloride (L-NAME) was from Sigma (Sigma-Aldrich, St. Louis, MO, USA). NAC and L-NAME were dissolved separately in phosphate-buffered saline just before use. For NAC treatment, culture medium was replaced with fresh culture medium containing 5 mM NAC and the cells were incubated overnight for approximately 15 h before EPS. For L-NAME treatment, the culture medium was replaced with fresh culture medium containing 2 mM L-NAME and cells incubated for 1 h before EPS.

### Quantitative reverse transcription polymerase chain reaction (qRT-PCR)

Total RNA including the mRNA fraction was isolated via lysis in 1 mL of Sepasol-RNA (Nakarai Tesque, Kyoto, Japan). One microgram of total RNA was reverse-transcribed using the PrimeScript RT Reagent Kit (TAKARA, Tokyo, Japan). Quantitative PCR was performed using the Fast SYBR Green Master Mix (Life Technology, Tokyo, Japan), 20 ng of cDNA, specific primers, and the qPCR System (CFX384 Touch; Bio-Rad, CA, USA) according to the manufacturer’s instructions.

The primer sequences are as follows. Nqo1; 5'-GGGTCGTCTTGGCAACCA-3' (Forward), 5'-CAGATGTTGAGGGAGGATCGTAA-3' (Reverse), Ho-1; 5'-CCTCACTGGCAGGAAATCATC-3' (Forward), 5'-CCTCGTGGAGACGCTTTACATA-3' (Reverse), GPx; 5'-CCTCAAGTACGTCCGACCTG-3' (Forward), 5'-CAATGTCGTTGCGGCACACC-3' (Reverse), G6pdx; 5'-ATGCAGGCCAACCGTCTATTCTA-3' (Forward), 5'-TCTCCACGATGATGCGGTTC-3' (Reverse), Gclm; 5'-AGTTGGAGCAGCTGTATCAGTGG-3' (Forward), 5'-TTTAGCAAAGGCAGTCAAATCTGG-3' (Reverse), Sod1; 5'-ATGGGTTCCACGTCCATCAGTA-3' (Forward), 5'-CATTGCCCAGGTCTCCAACA-3' (Reverse), Sod2; 5'-GAGAATCTCAGTGCTCACTCGTGTC-3' (Forward), 5'-GGAACCCTAAATGCTGCCAGTC-3' (Reverse), Gclc; 5'-ATCTGCAAAGGCGGCAAC-3' (Forward), 5'-ACTCCTCTGCAGCTGGCTC-3' (Reverse), Catalase; 5'-CGAGGGTCACGAACTGTGTCA-3' (Forward), 5'-GGTCACCCACGATATCACCAGATAC-3' (Reverse), cyclophilin; 5'-TGGAGAGCACCAAGACAGACA-3' (Forward), 5'-TGCCGGAGTCGACAATGAT-3' (Reverse).

The expression of the target mRNAs was normalized to cyclophilin mRNA. Relative quantification of gene expression was calculated based on the comparative CT (threshold cycle value) method (e) method (an), 20 Japan), 20cyclophilin gene). A comparison of gene expression in different samples was performed based on the differences in the ΔCT values for individual samples (ΔΔCT).

### Measurement of apoptosis via Annexin V/ Propidium Iodide (PI) analysis

EPS treated cells were maintained in a CO_2_ incubator for 2 hours after EPS. EPS-treated or untreated myotube cells were washed twice with PBS. After washing, binding buffer (1000 μl) containing FITC-Annexin-V (5 μl) and propidium iodide (PI; 10 μl) was added to the 3.5 cm dish according to the protocol for the Annexin V-FITC/PI kit (EBL Life Science, Tokyo, Japan). The samples were then incubated for 15 min in the dark at room temperature. After washing with PBS, the Annexin V-FITC/PI treated cells were evaluated under an epifluorescence microscope. Annexin V and PI-positive cells were counted in 20 HPF (high-power fields) from 3 sections prepared from cell cultured dishes for each group.

### Cell viability assessment using the MTT assay

C2C12 myotubes were grown in 6-well plates. After EPS treatment, 3-(4,5-dimethylthiazol-2-yl)-2,5-diphenyltetrazolium bromide stock (MTT; 50 μg/100 μL) was added to cells in each well for 4 h at 37°C. The MTT-containing medium was aspirated and 1000 μL DMSO was added to each well to dissolve the resulting formazan. These samples were then diluted 100-fold in DMSO and absorbance at 570 nm was read in a microplate reader (Bio-Rad Laboratories, Hercules, CA, USA).

### Statistical analysis

The data are presented as the mean ± the standard error of the mean (SEM). Differences were analyzed by Student’s t-test, p < 0.05 was considered statistically significant.

## Results

### ROS production and subsequent Nrf2 expression in C2C12 myotubes

**Fig 1:**
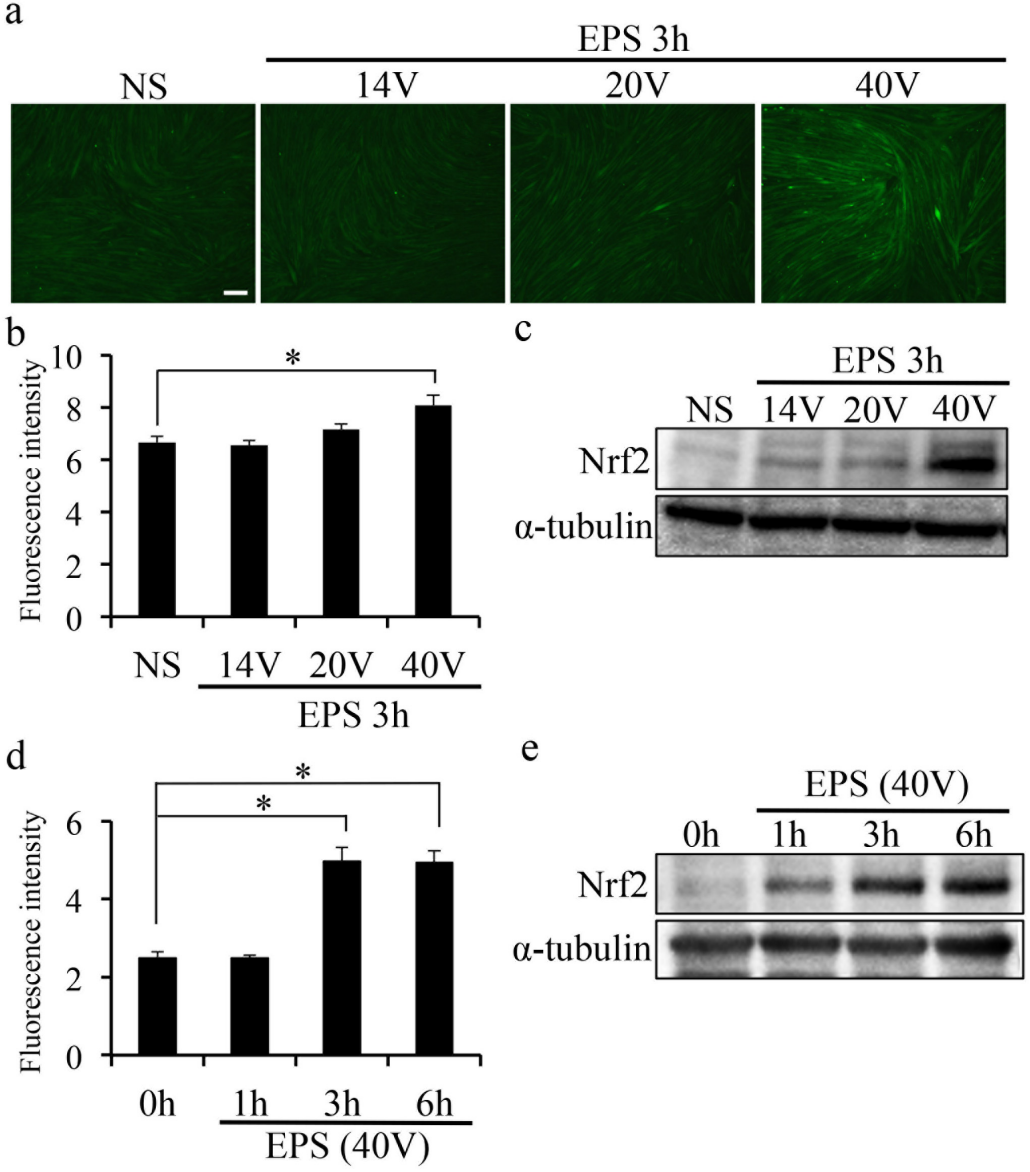
ROS production and Nrf2 expression induced in C2C12 myotubes by EPS. (a) Images show ROS/RNS production in C2C12 myotubes. EPS-treated C2C12 cells were loaded with DCFH-DA (20 μM) for 15 min. The static cells were then stimulated with EPS or left untreated for 3 h. Cells were washed with culture medium 3 times and live cells were imaged on an inverted fluorescence microscope. Scale bar = 100 μm. (b, d) Graph shows the fluorescence intensity of DCF/HPF in C2C12 myotubes. Fluorescence intensity of DCF was measured by fluorescence microscopy imaging (n = 3). (c, e) Protein expression levels assessed by western blotting are shown here. α-Tubulin was used as an internal standard for protein loading. *p < 0.05.

To accurately quantify ROS/RNS production, we loaded cells with the fluorescent probe 2′,7′-dichlorofluorescein diacetate (DCFH-DA) after EPS and subsequently measured fluorescence intensity values using an epifluorescence microscope and camera-based imaging analysis. DCFH-DA is converted to DCFH inside the cell, and when exposed to ROS/RNS, DCFH is oxidized to the fluorescent product 2′,7′-dichlorofluorescein (DCF). Fig 1 shows ROS/RNS generation after EPS in C2C12 myotubes. An EPS of 40 V for 3 h significantly increased DCF intensity in the myotubes, compared with in nonstimulated (NS) controls (40 V: 8.1 ± 0.39, NS: 6.5 ± 0.23, Fig 1a and 1b). However, an EPS for 14 V or 20 V did not induce ROS/RNS production in C2C12 myotubes (Fig 1a and 1b). In addition, Nrf2 protein levels were significantly increased by EPS for 40 V at 3 h (Fig 1c). In contrast, a shorter (1 h) EPS treatment (40 V) did not induce ROS/RNS production or increase Nrf2 protein levels (Fig 1d and 1e). However, EPS treatment for 3 h or more induced ROS/RNS production and increased Nrf2 protein levels (Fig 1d and 1e). These results suggest that higher intensity and chronic exposure of C2C12 myotubes to EPS was necessary to induce ROS/RNS production and increase Nrf2 protein expression.

### Nrf2 was essential for antioxidant and cytoprotective gene induction by EPS

**Fig 2:**
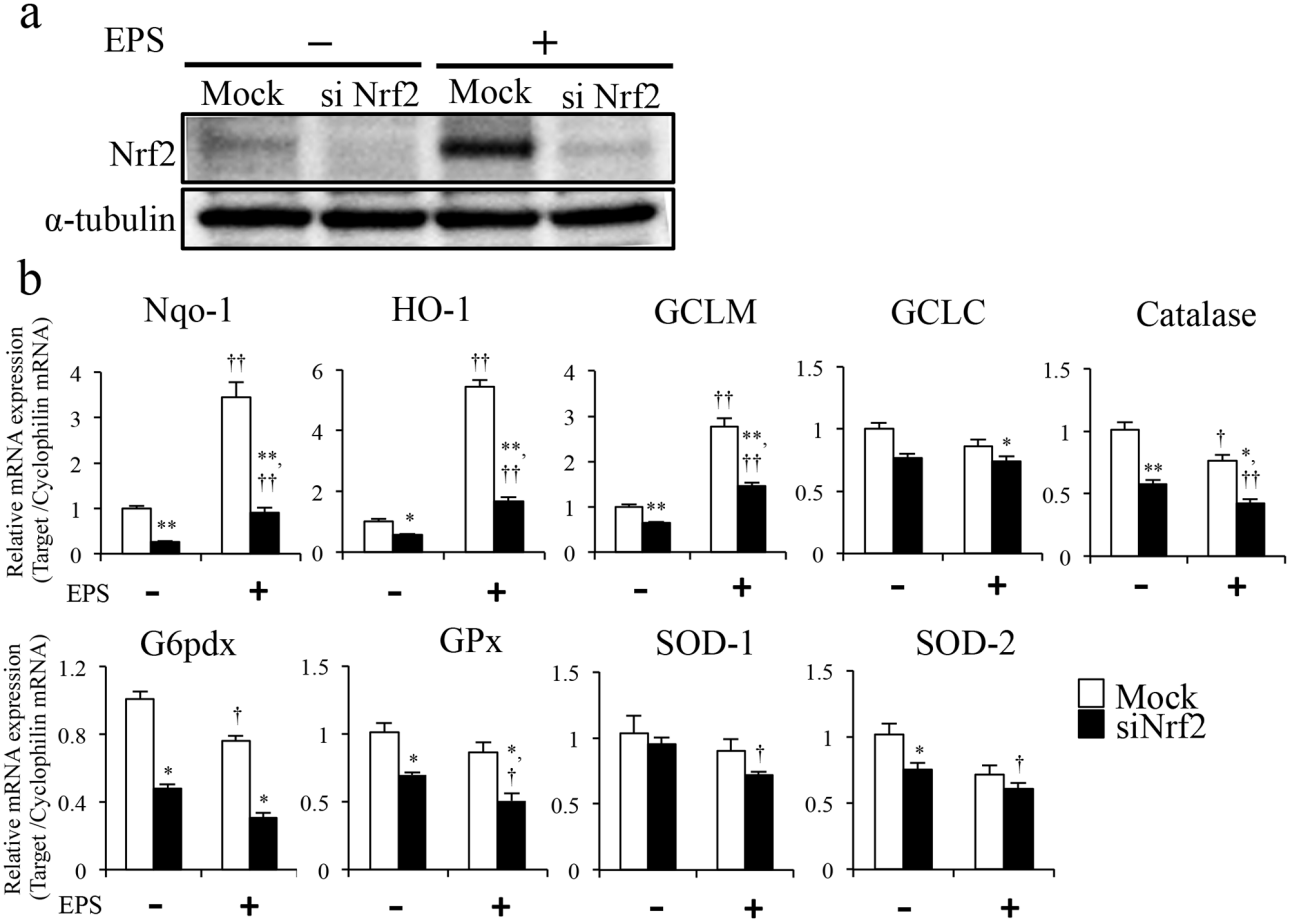
Attenuation of EPS-induced Nrf2 activation and Nrf2-related gene expression via Nrf2 knockdown in C2C12 myotubes. (a, b) C2C12 cells were transfected with siRNA against Nrf2 or a mock control. After 24 h transfection, cells were treated with EPS for 3 h. (a) Total cell lysate was analyzed by western blotting using an anti-Nrf2 antibody. (b) Expression levels of Nrf2-related genes were analyzed by quantitative PCR (n = 6). *p < 0.05, ** p < 0.001 vs. mock; †p < 0.05, ††p < 0.001 vs. non-EPS.

Nrf2 is a master transcriptional regulator of several antioxidant and cytoprotective genes [1]. To evaluate molecular pathways induced by EPS in C2C12 myotubes, we analyzed transcript levels of Nrf2-dependent antioxidant genes. Furthermore, to determine whether EPS induced Nrf2-dependent antioxidant genes via the Nrf2-Keap1 pathway, we developed an Nrf2 gene knockdown model in C2C12 cells using siRNA transfection. Interestingly, Nrf2 activation (Fig 2a) and mRNA levels of Nqo1, HO-1 and GCLM antioxidant genes were significantly upregulated in cells after EPS, compared with in NS controls (Nqo1: 3.4 ± 0.34-fold, HO-1: 5.4 ± 0.23-fold, GCLM: 2.8 ± 0.18-fold, Fig 2b). As shown in Fig 2, Nrf2 protein levels were markedly decreased following siRNA transfection (Fig 2a). Transfection with Nrf2-siRNA nearly abolished the induced levels of Nrf2 expression. Expression of Nqo-1, HO-1, GCLM, GCLC, catalase, GPx, G6pdx and SOD-2 were also substantially decreased (Fig 2b). Basal levels of Nqo1, HO-1 and GCLM mRNAs, as well as the levels of these genes induced by EPS, were reduced (Fig 2b).

**Fig 3:**
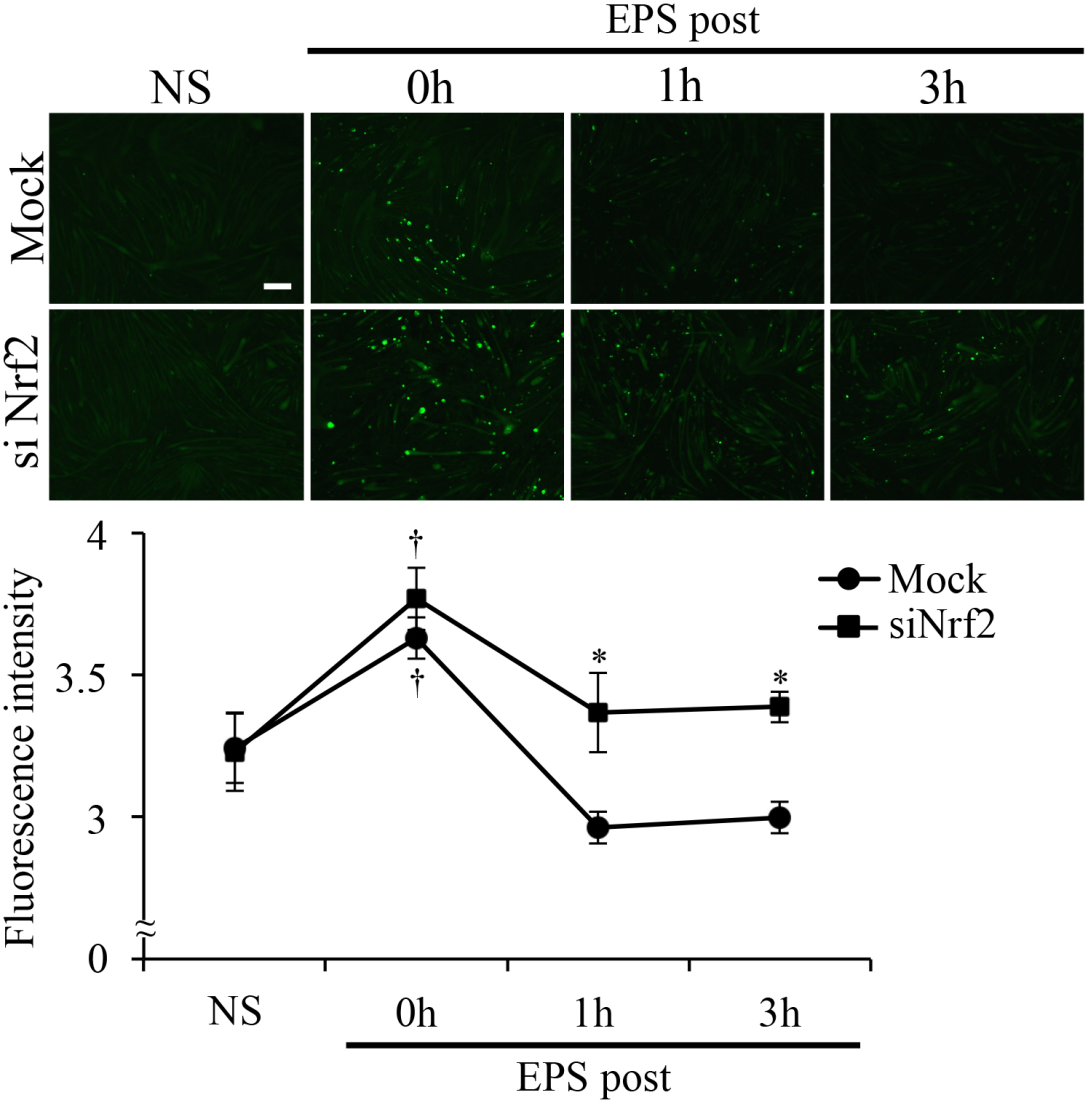
Effects of EPS-induced Nrf2 activation on ROS/RNS levels. EPS-treated and untreated C2C12 myotubes were loaded with DCFH-DA (20 μM) at 0 and after 1 and 3 h EPS. The graph shows the fluorescence intensity of DCF/HPF in C2C12 myotubes. Fluorescence intensity of DCF was measured using fluorescence microscopy imaging (n = 6). Scale bar = 100 μm. *p < 0.05 vs. mock; †p < 0.05 vs. non-EPS.

Our results suggest that Nrf2 was essential for reduction of EPS-induced ROS/RNS. Therefore, we measured ROS/RNS in mock- or siNrf2-transfected C2C12 myotubes after EPS. Mock-transfected C2C12 myotubes showed significantly increased ROS/RNS production (NS: 3.2 ± 0.084, EPS post-0 h: 3.6 ± 0.12) but these soon decreased (after 1 h EPS: 3.0 ± 0.054, after 3 h EPS: 3.0 ± 0.069, Fig 3). However, siNrf2-transfected C2C12 myotubes showed a markedly delayed decrease in ROS/RNS levels after stimulation of contraction, compared with the mock-transfected controls (NS: 3.2 ± 0.12, EPS post-0 h: 3.8 ± 0.10 after 1 h EPS: 3.4 ± 0.11, after 3 h EPS: 3.4 ± 0.065, Fig 3).

### Nrf2 protein and activated Nrf2-regulated gene induction were suppressed by antioxidants

**Fig 4:**
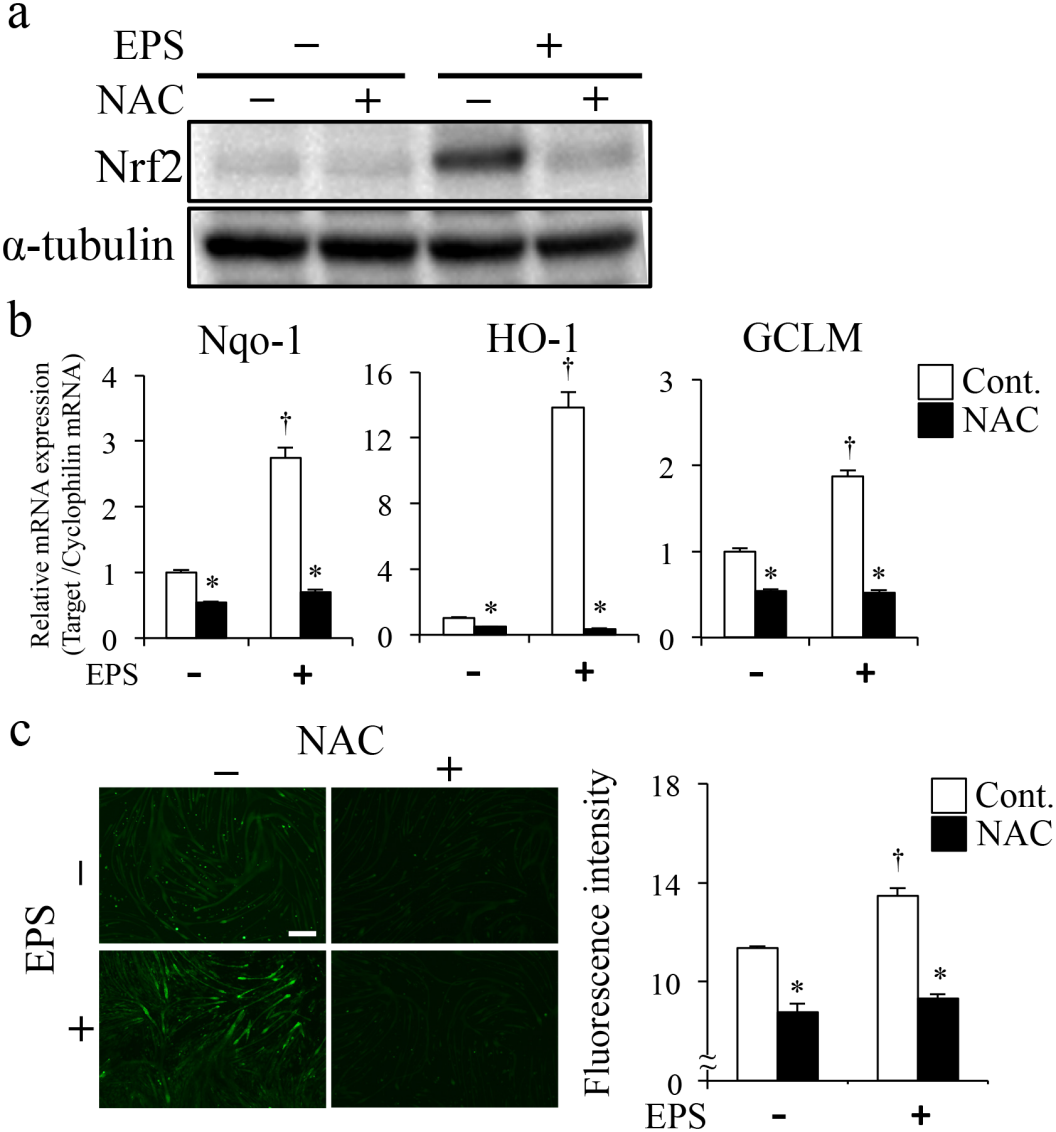
Effects of NAC treatment on EPS-induced Nrf2 levels and Nrf2-regulated gene expression. (a. b. c.) Overnight treatment with NAC (5 mM) followed by EPS for 3 h. (a) Total cell lysate was analyzed by western blotting using an anti-Nrf2 antibody. (b) Total RNA was prepared and relative expression levels of mRNA determined by quantitative PCR. (n = 6) (c) Images show ROS/RNS production in C2C12 myotubes. Scale bar = 100 μm. The graph shows fluorescence intensity of DCF/HPF in C2C12 myotubes. Fluorescence intensity of DCF was measured by fluorescence microscopy imaging (n = 6). *p < 0.05 vs. Control; †p < 0.05 vs non-EPS.

**Fig 5:**
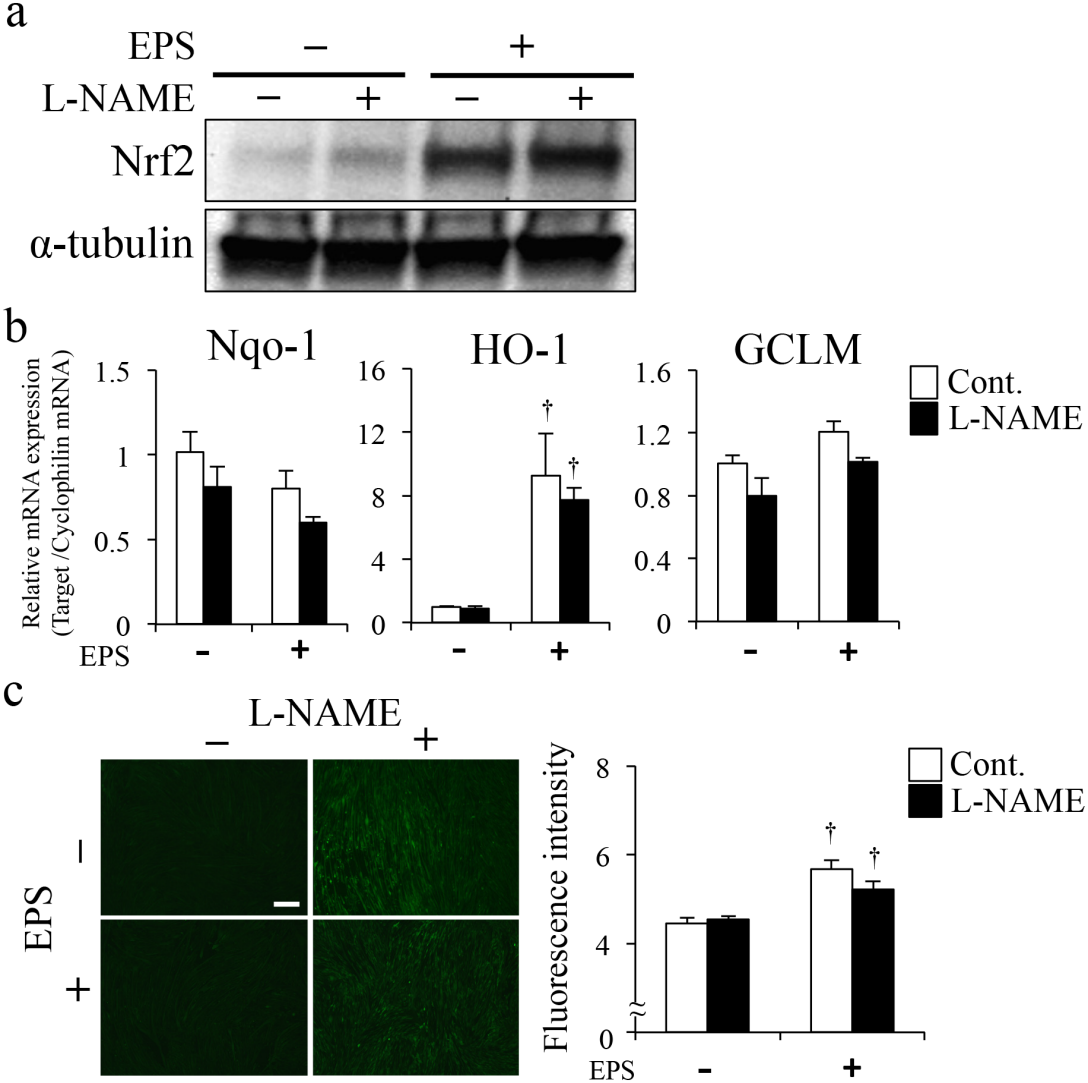
Effects of NOS inhibition on EPS-induced Nrf2 activation and Nrf2-regulated gene expression. (a, b, c) 1 hour with L-NAME (2 mM) followed by EPS for 3 h. (a) Total cell lysate was analyzed by western blotting using an anti-Nrf2 antibody. (b) Expression levels of Nrf2-regulated genes were analyzed by quantitative PCR (n = 3). (c) Images show ROS/RNS production in C2C12 myotubes. Scale bar = 100 μm. Graph shows the fluorescence intensity of DCF/HPF in C2C12 myotubes. Fluorescence intensity of DCF was measured by fluorescence microscopy imaging (n = 3). †p < 0.05 vs. non-EPS.

To further investigate the mechanisms by which Nrf2 was activated by EPS, we examined effects of the antioxidant N-acetylcysteine (NAC) on EPS-induced Nrf2 protein levels and activation, as indicated by expression of Nrf2-regulated genes. EPS induced increases in Nrf2 protein were completely diminished by treatment with 5 mM NAC (Fig 4a) and induction of Nrf2-regulated genes, such as NQO-1, HO-1 and GCLM, was also significantly suppressed (Fig 4b). These findings suggested that Nrf2 activation involved production of ROS/RNS elicited by EPS. C2C12 cells treated with NAC had significantly lower ROS/RNS levels (NS-Control: 11.9 ± 0.081, NS-NAC: 8.8 ± 0.028, EPS-Control: 13.5 ± 0.49, EPS-NAC: 9.3 ± 0.043, Fig 4c). We also investigated effects of the nitric oxide synthase (NOS) inhibitor L-NAME on expression of Nrf2-regulated genes induced by EPS and found that inhibition of NO production did not affect gene expression (Fig 5a and 5b). Furthermore, L-NAME treatment in C2C12 cells did not inhibit production of ROS/RNS, as detected by fluorescence of DCF, the broadly specific indicator (NS-Control: 4.5 ± 0.12, NS-L-NAME: 4.5 ± 0.067 EPS-Control: 5.7 ± 0.19 EPS-L-NAME: 5.2 ± 0.17, Fig 5c). These results confirmed that, in response to EPS, ROS, not RNS, mediates Nrf2 activation.

### Nrf2 activation inhibits EPS-related myotubes wasting

**Fig 6:**
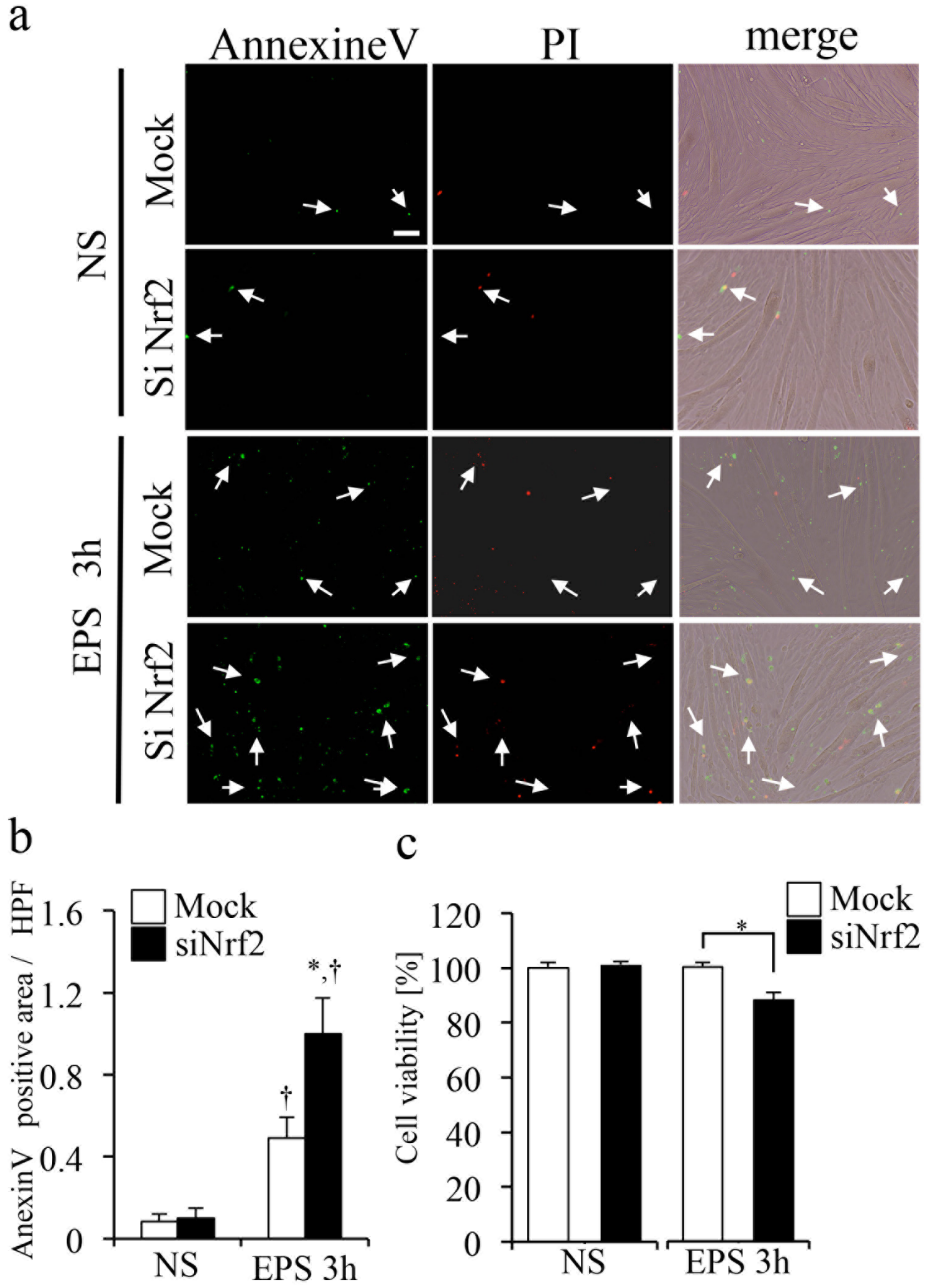
Inhibition of EPS-induced cell wasting in C2C12 myotubes by Nrf2 activation. (a) Images show immunocytochemical staining of Annexin V and propidium iodide (PI) staining in EPS-treated and untreated C2C12 myotubes. Annexin V positive signals are indicated with arrows. (b) The graph shows fluorescence intensity of Annexin V/HPF in C2C12 myotubes. Annexin V expression was measured by fluorescence microscopy imaging (n = 6). (c) Graph shows the cell viability of non-EPS or EPS treated C2C12 myotubes (n = 6) as determined by the MTT assay. *p < 0.001 vs. mock-transfected; †p < 0.05 vs. non-EPS.

Increased ROS levels after muscle contraction induced apoptosis in muscle tissue [11]. Therefore, we examined ROS-related apoptosis in EPS-treated C2C12 myotubes (Fig 6). EPS significantly increased expression of the apoptosis marker Annexin V in EPS-treated C2C12 cells (NS-mock: 0.085 ± 0.037, EPS-mock: 0.49 ± 0.10, Fig 6a and 6b). Furthermore, knockdown of Nrf2 with siRNA treatment markedly increased Annexin V expression, compared with mock transfection (NS-siNrf2: 0.10 ± 0.049, EPS-siNrf2: 1.00 ± 0.18, Fig 6a and 6b). Based on the MTT assay results, knockdown of Nrf2 with siRNA transfection significantly decreased cell viability after EPS stimulation, compared with mock transfection (Fig 6c).

## Discussion

In this study, we showed that electrical stimulation increased ROS and caused increased levels of Nrf2 protein. Our results also demonstrated that muscle contraction by EPS induced gene expression in C2C12 myotubes via the Nrf2-Keap1 pathway. We showed that mRNA expression of the Nrf2-induced genes NQO1, HO-1 and GCLM was increased by EPS-induced muscle contraction and significantly suppressed by Nrf2 knockdown. Next, using an NOS inhibitor and an antioxidant we found that the antioxidant NAC significantly attenuated Nrf2 activation by EPS-induced muscle contraction, implicating oxidative stress in this response; in contrast, NO production did not affect Nrf2 activation in our system. We further demonstrated that abrogation of Nrf2 in combination with EPS-induced oxidative stress induced apoptosis in C2C12 myotubes.

Pan et al. demonstrated ROS release after EPS (45 V, 5 Hz) stimulation in vitro [12]. This is consistent with our observation that C2C12 myotubes exhibited a large increase in ROS production during longer exposure to comparable intensities of EPS (Fig 1). These results suggested that EPS-induced contraction generated ROS/RNS in C2C12 cells and that ROS/RNS production depended on the intensity and time of muscle contraction. Previous studies demonstrated that high intensity exercise induced ROS production [13,14]. Our findings suggest that EPS at a high-voltage intensity may have similar effects to those of high intensity exercise. Fig 1 also showed increased Nrf2 protein expression in response to EPS. Under basal conditions, Nrf2 is primarily localized in a complex with Keap1 and is degraded by the ubiquitin proteasome pathway. However, Nrf2 liberated from Keap1 under conditions of oxidative stress is stabilized and can accumulate in the cell. Thus, the data in Fig 1, including the higher protein levels, suggested that Nrf2 was activated by muscle contraction induced by EPS. These findings suggest that NRF2 is relevant to endurance or to high-intensity exercise. Fig 2 showed that EPS-induced ROS/RNS production drove Nrf2 activation and increased expression of Nrf2 regulated genes (Fig 2). Furthermore, we clearly demonstrated that Nrf2 activation was essential to reducing ROS/RNS production after EPS (Fig 3). Thus, our results suggest that Nrf2 is an important factor for a system of protection against oxidative stress during muscle contraction. Regulation of Nrf2 signaling is believed to preserve redox homeostasis and protect the structure and function of cells [1]. Excessive production of ROS/RNS causes oxidative stress, a key signal for the onset of several musculoskeletal diseases [15–17]. Some studies have reported that increased oxidative stress decreased skeletal muscle performance during exercise [7,10]. Therefore, Nrf2 activation may be considered a potential positive regulator of muscle function during exercise. Furthermore, exercise-induced ROS production in skeletal muscle was shown to be an important factor for muscle adaptation during exercise training [18–20]. Such studies suggested that responses to oxidative stress are involved in adaptation during intense exercise. Thus, our findings suggest that Nrf2 signaling is also an important factor for exercise adaptation in skeletal muscle.

The effects of NAC treatment showed that Nrf2 activation was induced by the increased ROS/RNS production during EPS (Fig 4). Most previous reports have assumed that skeletal muscle serves as the major source of free radicals and ROS generation during muscle contraction [5,6]. However, Jackson et al. discussed several other potential production sites for NO, as well as ROS, in muscle following damage [7,11]. Therefore, we investigated effects of NO production on Nrf2 activation and Nrf2-regulated gene expression. We found that Nrf2 activation was an NO-independent response to EPS-induced muscle contraction. These results indicated that ROS, for example superoxide, and not NO, was involved in the EPS-induced activation of Nrf2 (Fig 5).

Superoxide dismutases (SOD) are enzymes that catalyze the dismutation of the superoxide (O_2_^-^) radical into either molecular oxygen (O_2_) or hydrogen peroxide (H_2_O_2_) [21–23]. SOD1 requires copper and zinc as cofactors and is primarily located in the cytosol and the mitochondrial intermembrane space. SOD2 uses manganese as a cofactor and is located in the mitochondrial matrix. These enzymes are known to be important components of the antioxidant response in muscle during exercise. However, we showed that expression of SOD1 and SOD2 mRNA was not affected by EPS (Fig 3). In addition, expression of neither protein was affected by EPS (data not shown). Thus, these findings suggest that the response to oxidative stress after EPS was not dependent on SOD expression.

Several studies have reported that oxidative stress and other pathological conditions are strongly correlated with protein degradation and increased cell death [11,24–27]. As shown in Figs 1 and 3, EPS induced excessive ROS production in contracting C2C12 myotubes. Therefore, we analyzed apoptotic pathways under basal and EPS-stressed conditions in wild type and Nrf2-knockdown (siNrf2) C2C12 myotubes. We demonstrated that Nrf2 knockdown markedly increased apoptosis in EPS-treated C2C12 myotubes (Fig 6). We suggested that metabolism of ROS resulting from Nrf2 activation protected the myotubes from EPS-induced apoptosis. Furthermore, cell viability in siNrf2 myotubes after EPS was significantly lower than that in the mock transfected myotubes (Fig 6). Thus, these results indicated that Nrf2 signaling might prevent muscle wasting after excessive contraction.

In conclusion, our findings suggest that Nrf2 is a key player promoting cytoprotection in skeletal muscle against ROS produced during muscle contraction.

## References

[1] Itoh K, Mimura J, Yamamoto M. Discovery of the negative regulator of Nrf2, Keap1: a historical overview. Antioxid Redox Signal. 2010; 13: 1665–1678. 10.1089/ars.2010.3222 20446768

[2] Itoh K, Wakabayashi N, Katoh Y, Ishii T, Igarashi K, Engel JD, et al Keap1 represses nuclear activation of antioxidant responsive elements by Nrf2 through binding to the amino-terminal Neh2 domain. Genes Dev. 1999; 13: 76–86. 9887101 10.1101/gad.13.1.76 PMC316370

[3] Kobayashi M, Itoh K, Suzuki T, Osanai H, Nishikawa K, Katoh Y, et al Identification of the interactive interface and phylogenic conservation of the Nrf2-Keap1 system. Genes Cells. 2002; 7: 807–820. 12167159 10.1046/j.1365-2443.2002.00561.x

[4] Blair SN, Cheng Y, Holder JS. Is physical activity or physical fitness more important in defining health benefits? Med Sci Sports Exerc. 2001; 33: 379–399. 10.1097/00005768-200106001-00007 11427763

[5] Crespo CJ, Palmieri MR, Perdomo RP, McGee DL, Smit E, Sempos CT, et al The relationship of physical activity and body weight with all-cause mortality: results from the Puerto Rico Heart Health Program. Ann Epidemiol. 2002; 12: 543–552. 12495827 10.1016/s1047-2797(01)00296-4

[6] Jackson MJ. Control of reactive oxygen species production in contracting skeletal muscle. Antioxid Redox Signal. 2011; 15(9): 2477–2486. 10.1089/ars.2011.3976 21699411 PMC3176346

[7] Jackson MJ. Free radicals generated by contracting muscle: By-products of metabolism or key regulators of muscle function? Free Rad Biol Med. 2008; 44: 132–141. 10.1016/j.freeradbiomed.2007.06.003 18191749

[8] Powers SK, Jackson MJ. Exercise-induced oxidative stress: Cellular mechanisms and impact on muscle force production. Physiol Rev. 2008; 88: 1243–1276. 10.1152/physrev.00031.2007 18923182 PMC2909187

[9] McArdle A, Pattwell D, Vasilaki A, Griffiths RD, Jackson MJ. Contractile activity-induced oxidative stress: Cellular origin and adaptive responses. Am J Physiol. 2001; 280: 621–627. 10.1152/ajpcell.2001.280.3.C621 11171582

[10] Pattwell DM, McArdle A, Morgan JE, Patridge TA, Jackson MJ. Release of reactive oxygen and nitrogen species from contracting skeletal muscle cells. Free Radic Biol Med. 2004; 37: 1064–1072. 15336322 10.1016/j.freeradbiomed.2004.06.026

[11] Jackson MJ. Redox regulation of skeletal muscle. IUBMB Life. 2008; 60(8): 497–501. 10.1002/iub.72 18629903

[12] Pan H, Xu X, Hao X, Chen Y. Changes of myogenic reactive oxygen species and interleukin-6 in contracting skeletal muscle cells. Oxid Med Cell Longev. 2012; 2012: 145418 10.1155/2012/145418 22666517 PMC3361309

[13] Aldred S. Oxidative and nitrative changes seen in lipoproteins following exercise. Atherosclerosis. 2007; 192: 1–8. 17349647 10.1016/j.atherosclerosis.2007.02.001

[14] Andrade FH, Reid MB, Westerblad H. Contractile response of skeletal muscle to low peroxide concentrations: myofibrillar calcium sensitivity as a likely target for redox-modulation. FASEB J. 2001; 15: 309–311. 11156946 10.1096/fj.00-0507fje

[15] Aoi W, Sakuma K. Oxidative stress and skeletal muscle dysfunction with aging. Curr Aging Sci. 2011; 4: 101–109. 21235498 10.2174/1874609811104020101

[16] Gianni P, Jan KJ, Douglas MJ, Stuart PM, Tarnopolsky MA. Oxidative stress and the mitochondrial theory of aging in human skeletal muscle. Exp Gerontol. 2004; 39: 1391–1400. 15489062 10.1016/j.exger.2004.06.002

[17] Peleli M, Aggeli IK, Matralis AN, Kourounakis AP, Beis I, Gaitanaki C. Evaluation of two novel antioxidants with differential effects on curcumin-induced apoptosis in C2 skeletal myoblasts; involvement of JNKs. Bioorg Med Chem. 2015; 23(3): 390–400. 10.1016/j.bmc.2014.12.046 25577709

[18] Powers SK, Duarte J, Kavazis AN, Talbert EE. Reactive oxygen species are signaling molecules for skeletal muscle adaptation. Exp Physiol. 2010; 95: 1–9. 10.1113/expphysiol.2009.050526 19880534 PMC2906150

[19] Gomez-Cabrera MC, Domenech E, Romagnoli M, Arduini A, Borras C, Pallardo FV, et al Oral administration of vitamin C decreases muscle mitochondrial biogenesis and hampers training-induced adaptations in endurance performance. Am J Clin Nutr. 2008; 87: 142–149. 18175748 10.1093/ajcn/87.1.142

[20] Ristow M, Zarse K, Oberbach A, Klöting N, Birringer M, Kiehntopf M, et al Antioxidants prevent health-promoting effects of physical exercise in humans. Proc Natl Acad Sci U S A. 2009; 106(21): 8665–8670. 10.1073/pnas.0903485106 19433800 PMC2680430

[21] Ji LL. Antioxidant signaling in skeletal muscle: a brief review. Exp Gerontol. 2007; 42(7): 582–593. 17467943 10.1016/j.exger.2007.03.002

[22] Vasilaki A, Jackson MJ. Role of reactive oxygen species in the defective regeneration seen in aging muscle. Free Radic Biol Med. 2013; 65: 317–323. 10.1016/j.freeradbiomed.2013.07.008 23851030 PMC3859734

[23] Powers SK, Hamilton K. Antioxidants and exercise. Clin Sports Med. 1999; 18(3): 525–536. 10410839 10.1016/s0278-5919(05)70166-6

[24] Powers SK, Kavazis AN, McClung JM. Oxidative stress and disuse muscle atrophy. J Appl Physiol. 2007; 102(6): 2389–2397. 17289908 10.1152/japplphysiol.01202.2006

[25] Miller CJ, Gounder SS, Kannan S, Goutam K, Muthusamy VR, Firpo MA, et al Disruption of Nrf2/ARE signaling impairs antioxidant mechanisms and promotes cell degradation pathways in aged skeletal muscle. Biochim Biophys Acta. 2012; 1822(6): 1038–1050. 10.1016/j.bbadis.2012.02.007 22366763

[26] Lee YH, Kim DH, Kim YS, Kim TJ. Prevention of oxidative stress-induced apoptosis of C2C12 myoblasts by a Cichorium intybus root extract. Biosci Biotechnol Biochem. 2013; 77(2): 375–377. 23391909 10.1271/bbb.120465

[27] Niess AM, Simon P. Response and adaptation of skeletal muscle to exercise the role of reactive oxygen species. Frontiers in Bioscience. 2007; 12: 4826–4838. 17569613 10.2741/2431

